# The Agassiz Memorial

**Published:** 1874-06

**Authors:** 


*The Agassiz Memorial—Teachers and Pupils Fund*—"Louis Agassiz, Teacher.—This was the heading of his simple will; this was his chosen title; and it is well known throughout this country, and in other lands, how much he has done to raise the dignity of the profession, and to improve its methods. His friends, the friends of education, propose to raise a memorial to him, by placing upon a strong and enduring basis the work to which he devoted his life, the Museum of Comparative Zoölogy, which is at once a collection of natural objects, rivalng the most celebrated collections of the Old World, and a school open to all the teachers of the land.

It is proposed that the teachers and pupils of the whole country take part in this memorial, and that from the birthday of Agassiz, the 28th day of May, 1874, they shall each contribute something, however small, to the TEACHERS AND PUPILS' MEMORIAL FUND, in honor of LOUIS AGASSIZ; the fund to be kept separate, and the income to be applied to the expenses of the Museum.

John Eaton, Commissioner of Education, Washington, D. C; Joseph Henry, Secretary of the Smithsonian Institution, Washington, D. C; Joseph White, Secretary of the Board of Education of Massachusetts, Boston; W. T. Harris, Superintendent, Public Schools, St. Louis, Mo.; Edward J. Lowell, Boston John S. Blatchford, Boston; Jas. M. Barnard, Treasurer Teachers and Pupils Fund, Boston.

All communications and remittances for the "Teachers' and Pupils' Fund" of the "Agassiz Memorial," may be sent to the Treasurer, Jas. M. Barnard, Room 4, No. 13 Exchange Street, Boston.

---

### MONTHLY SUMMARY.

